# Translation and validation of a Bahasa Malaysia (Malay) version of the Multidimensional Assessment of Interoceptive Awareness (MAIA)

**DOI:** 10.1371/journal.pone.0231048

**Published:** 2020-04-01

**Authors:** Jennifer Todd, David Barron, Jane E. Aspell, Evelyn Kheng Lin Toh, Hanoor Syahirah Zahari, Nor Azzatunnisak Mohd. Khatib, Viren Swami

**Affiliations:** 1 School of Psychology and Sport Science, Anglia Ruskin University, Cambridge, United Kingdom; 2 Centre for Psychological Medicine, Perdana University, Serdang, Malaysia; Universiti Sains Malaysia, MALAYSIA

## Abstract

**Objectives:**

The 32-item Multidimensional Assessment of Interoceptive Awareness (MAIA) is a widely-used measure of multidimensional interoception. In the present study, we examined the psychometric properties of a Bahasa Malaysia (Malay) translation of the MAIA.

**Methods:**

An online sample of 815 Malaysian Malays (women *n* = 403) completed a novel translation of the MAIA. Validated measures of trait mindfulness and self-esteem were also completed to facilitate a preliminary assessment of convergent validity.

**Results:**

Exploratory factor analysis indicated that the MAIA items reduced to a 19-item, 3-factor model. The 3-factor model was further tested using confirmatory factor analysis (CFA) alongside the parent 8-factor model. Both models had good fit on some indices, but less-than-ideal fit on other indices. The 3-factor model evidenced comparatively better fit, with fit indices being adequate following modification. Multi-group CFA indicated both the 3-factor model and the 8-factor model had full strict invariance across sex. However, evidence for construct and convergent validity was mixed.

**Conclusions:**

Overall the 3-dimensional Malay MAIA was demonstrated to be both internally consistent and invariant across sex, but further evidence of construct and convergent validity is required. Issues that affect the dimensionality of MAIA scores in the present and extant work are discussed in conclusion.

## Introduction

The term *interoception* refers to a collection of processes through which the physiological state of the body is communicated to the brain [1]. Internal organs–such as the heart, lungs, and stomach–produce signals that constantly indicate their present condition. The nervous system then detects, interprets, and integrates this information to generate a continuous account of the body’s internal state [1, 2]. Self-reported detections of interoceptive stimuli, measured via questionnaires, are commonly referred to as *interoceptive awareness* (IA; cf. [3]). IA is, in itself, a multidimensional construct [4], encompassing appraisals and beliefs surrounding interoceptive stimuli, the regulation of attention toward interoceptive stimuli, and behavioural responses [4, 5].

To capture all of these aspects of IA, Mehling and colleagues [6] developed the Multidimensional Assessment of Interoceptive Awareness (MAIA). The authors utilised an extensive, mixed-methods process, which included reviewing the current literature on multidimensional conceptual frameworks, evaluating existing instruments [5], analysing focus group responses to concepts and items, pre-testing items for semantic validity, and exploratory cluster and confirmatory factor analyses. The resulting 32-item measure comprises eight subscales which assess: the self-perceived tendency to notice positive, negative, and neutral bodily sensations (Noticing subscale, 4 items); the inclination to either acknowledge or ignore sensations of discomfort/pain (Not-Distracting subscale, 3 items); the extent to which sensations of discomfort/pain provoke feelings of worry (Not-Worrying subscale, 3 items); the self-perceived degree to which attention towards bodily sensations can be to controlled and sustained (Attention Regulation subscale, 7 items); the degree of awareness regarding relations between bodily and emotional states (Emotional Awareness subscale, 5 items); the tendency to alleviate distress through use of attention toward bodily sensations (Self-Regulation subscale, 4 items); the tendency to actively ‘listen’ to bodily sensations for insight (Body Listening subscale, 3 items); and, finally, the extent to which bodily sensations are considered to be ‘safe’ and ‘trustworthy’ information sources (Trusting subscale, 3 items).

In the parent study, construct validity for the MAIA was established through an array of correlations with scores on measures of mindful attention and body awareness, measures of trait anxiety and anxiety in response to pain, and measures of emotional regulation. MAIA scores were also demonstrated to distinguish between groups of participants with differing levels of experience with mindfulness and body awareness practices. In particular, participants with greater levels of experience had higher scores across all subscales and differences were statistically significant for the Noticing and Attention Regulation subscales. Within the wider literature, the multidimensional nature of the MAIA has proved a useful contribution to knowledge (for a review, see [4]). For example, use of the MAIA has allowed researchers to identify specific facets of interoception that can be modified through training interventions [7–9].

The MAIA has, to date, also been translated into over 20 languages, and numerous examinations of the dimensionality of MAIA scores in different linguistic and sociocultural groups have been conducted (for a summary, see Table 1). Though most studies have retained the 8-factor model some have struggled with the estimation of the Not-Distracting and Not-Worrying subscales [10–12]. In particular, Items 8 and 10 from the Not-Worrying subscale have been consistently problematic due to low factor loadings or loading onto other factors [10–13]. Similar difficulties have been encountered with Item 19 from the Emotional Awareness subscale, which has been found to load onto Body Listening [7, 10], and Item 4 from the Noticing subscale, which has been found to load onto Emotional Awareness [10, 12]. Consequently, some translational studies have proposed an 8-factor model with a reduced number of items [10, 12]. Other translational studies have failed to replicate the parent model, instead finding that MAIA scores reduce to seven [14] or six [15–18] dimensions. Finally, other researchers have elected to deliberately reduce the number of MAIA dimensions to overcome difficulties with high correlation between the MAIA subscales and increase statistical power [19, 20].

**Table 1:** Examinations of the factorial validity of the multidimensional assessment of interoceptive awareness.

Reference (organised by date order)	Language	Country	Sample type	*N*	Data reduction method	Extraction Criterion	Dimensionality	Fit Indices (Final Model)	Cronbach *α* values
[6] Mehling et al. (2012)	English	United States	Community (varying experience of mindfulness-based practises)	325 adults (*M* = 48 years, 79% women). Less experienced (38%), highly experienced (62%)	Exploratory cluster analyses and CFA	Iterative process; λ > 1.0	Eight dimensions (32 items)	χ^2^(436) = 927.3, *p* < .001; CFI = .89; TLI = .87; RMSEA = .60 (90% CI .055-.066); SRMR = .06	NT = .69 ND = .66 NW = .67 AR = .87 EA = .82 SR = .83 BL = .82 TR = .79
[71] Mehling et al. (2013)	English	United States	Clinical (current or past back-pain)	435 adults (*M* = 54 years, 53% women)	Exploratory cluster analyses CFA	Iterative process; λ > 1.0	Seven dimensions (29 items, Not-Distracting excluded)	CFI = .88; RMSEA = .07	NT = .74 ND = .48 NW = .58 AR = .88 EA = .90 SR = .86 BL = .83 TR = .78
[7] Bornemann et al. (2015)	German	Germany	Community	1076 adults (≥18 years, 68% women)	EFA and CFA	λ > 1.0	Eight dimensions (32 items)	CFI = .90; RMSEA = .06	NT = .76, ND = .56, NW = .65, AR = .89, EA = .86, SR = .84, BL = .84, TR = .86
[10] Calì et al. (2015)	Italian	Italy	College	321 adults (≥19 years, 91% women)	EFA and CFA	λ > 1.0	Eight dimensions (29 items), with modifications.	χ^2^(349) = 408.99, *p* = .015; CFI = .97; RMSEA = .02 (90% CI .01-.03); SRMR = .06	NT = .68, ND = .53, NW = .59, AR = .75, EA = .79, SR = .75, BL = .74, TR = .80
[12] Valenzuela-Moguillansky, & Reyes-Reyes (2015)	Spanish	Chile	Community and college	470 adults (≥18 years, 77% women)	EFA and CFA	Iterative process, multiple EFAs conducted to find a solution with item loadings > .30.	Eight dimensions (30 items), with modifications.	SBχ^2^(371) = 659.78, *p* < .001; CFI = .92; TLI = .91; RMSEA = .06 (90% CI = .05-.06); SRMR = .06	NT = .64, ND = .49, NW = .40, AR = .86, EA = .82, SR = .85, BL = .83, TR = .86
[17] Gim et al. (2016)	Korean	Korea	Community (varying levels of experience with mindfulness-based activities)	518 adults	EFA and CFA	Parallel analysis	Six dimensions (32 items), with modifications.	χ^2^(436) = 793.52; CFI = .92; TLI .90; RMSEA = .06 (90% CI .05-.07)	.80 - .90
[72] Brown et al. (2017)	English	United States	Clinical (eating disorder diagnosis)	376 patients (182 adults and 194 adolescents; 94% women)	EFA and CFA	Not specified	Eight dimensions (32 items)	CFI = .88; TLI = .83; RMSEA = .07 (90% CI = .07-.08)	NT = .76, ND = .67, NW = .62, AR = .91, EA = .84, SR = .89, BL = .89, TR = .92
[13] Lin et al. (2017)	Chinese	Taiwan	Convenience sample of adults with experience in mindfulness-based activities	294 adults (≥20 years, 70% women). Less experienced (*n* = 218), highly experienced (*n* = 76)	CFA	N/A	Eight dimensions (32 items), with modifications	χ^2^(434) = 978.27; *p* < .001; CFI = .96; RMSEA = .07 (95% CI = .06-.07); GFI = .83	NT = .76, ND = .58, NW = .46, AR = .85, EA = .88, SR = .81, BL = .87, TR = .86
[20] Stern et al. (2017)	English	United States	Community	19 healthy adults	EFA	λ > 1.0	Three dimensions (32 items)	Not reported.	Not reported.
[73] Abbasi et al. (2018)	Persian	Iran	College	425 adults (56.7% women)	EFA	λ > 1.0	Eight dimensions (32 items)	Published in Persian	.53 - .83
[15] Baranauskas et al. (2018)	Lithuanian	Lithuania	College	386 adults (≥17 years, 49% women)	CFA	N/A	Six dimensions (25 items)—Not-Distracting and Noticing discarded prior to CFA due to low *α*.	χ^2^(260) = 760.91, *p* < .001; CFI = .85; IFI = .85; RMSEA = .07; SRMR = .07	NT = < .60 ND = < .50 NW = .63 AR = ≥ .70 EA = ≥ .70 SR = ≥ .70 BL = ≥ .70 TR = ≥ .70
[14] Machorrinho et al. (2018)	Portuguese	Portugal	College	490 adults (≥18 years, 58% women)	EFA and CFA	Not specified.	Seven dimensions (33 items)–Body Listening was eliminated	χ^2^ = 1206.9; *p* < .001; CFI = .82; RMSEA = .07 (90% CI = .07 - .08); SRMR = .07	NT = .61, ND = .81, NW = .74, AR = .86, EA = .80, SR = .87, TR = .81
[18] Shoji et al. (2018)	Japanese	Japan	College	390 adults (*M* = 20.3 years, 68% women)	EFA (CFA mentioned but not reported)	λ > 1.0	Six dimensions (25 items)–Not Worrying and Self-Regulation were eliminated	Not reported	NT = .74, ND = .67, AR = .87, EA = .85, BL = .84, TR = .83
[26] Mehling et al. (2018)	English	United Kingdom	Community	1090 adults (≥18 years, 47% women)	Exploratory cluster analysis and CFA	Iterative process; λ > 1.0	Eight dimensions (37 items)	χ^2^(601) = 1597.7; *p* < .001; CFI = .86; TLI = .85; RMSEA = .06 (90% CI = .05-.06); SRMR = .06.	NT = .64 ND = .74 NW = .67 AR = .83 EA = .79 SR = .79 BL = .80 TR = .83
[19] Mul et al. (2018)	English	United Kingdom	Clinical (autism diagnosis) and community	52 participants (*M* = 25.9 years, 14 women)	Multi-dimensional scaling	Not reported	Three dimensions (32 items)	Normalised raw stress = 0.035	Not reported
[11] Reis (2019)	German	Germany	Community	320 adults (*M* = 41.3 years, 70% women)	CFA, ESEM, and BSEM	N/A	Eight dimensions (32 items)	CFA: χ^2^(436) = 939.4; *p* < .001; CFI = .90; TLI = .89; RMSEA = .06; SRMR = .06 BSEM: PPP = .78; DIC = 22,189.5	NT = .74 ND = .64 NW = .67 AR = .91 EA = .84 SR = .88 BL = .86 TR = .86
[16] Fujino (2019)	Japanese	Japan	College and community	268 adults (*M* = 19.6 years, 53% women)	CFA	N/A	Six dimensions (25 items, as in Shoji et al., 2018)	χ^2^(159) = 684.2; *p* < .001; CFI = .98; TLI = .98; RMSEA = .078 (90% CI = .071-.085); SRMR = .067.	NT = .78, ND = .62, AR = .87, EA = .84, BL = .82, TR = .80

EFA = Exploratory Factor Analysis, CFA = Confirmatory Factor Analysis, ESEM = Exploratory Structural Equation Modelling, BSEM = Bayesian Structural Equation Modelling, CFI = Comparative fit index, TLI = Tucker-Lewis index, RMSEA = Steiger-Lind root mean square error of approximation, SRMR = standardised root mean square residual, PPP = posterior predictive *p* value, DIC = deviance information criterion, IFI = incremental fit index, NT = Noticing, ND = Not-Distracting, NW = Not-Worrying, AR = Attention Regulation, EA = Emotional Awareness, SR = Self-Regulation, BL = Body Listening, TR = Trusting.

A number of additional methodological issues are noteworthy across the available literature. First, internal consistency has been routinely suboptimal. In the parent study, coefficients were below acceptable thresholds (α > .70) for the Noticing, Not-Worrying, and Not-Distracting subscales, and similar issues have been noted for all of the available validations of the measure (see Table 1). Furthermore, it is likely that internal consistency has been underestimated due to reliance on Cronbach’s α. This is because one assumption of α is the need for a *τ*-equivalent model [21], where true score variance is assumed to be equal across all items (i.e., factor loadings are equal). As this has not been the case within the available MAIA studies, the use of ω [22] is likely to provide a more reliable estimate [11, 21].

Second, model fit indices have been adequate at best and often relatively poor (see Table 1). Again, this appears to stem from an initial poor fit within the parent study [6]. While it is important that fit indices are not relied upon inflexibly when judging model fit [23–25], the prevalence of the issue suggests that model re-specification may be necessary. Indeed, in response to some of the aforementioned problems, Mehling and colleagues [26] recently published an updated version of the MAIA (the MAIA-2). The authors sought to improve internal consistency within the Not-Distracting and Not-Worrying subscales through the addition of 5 new items. Though internal consistency was improved for the Not-Distracting subscale (α = .74, Δα = .08), the Noticing and Not-Distracting subscales remained suboptimal (α < .70), and model fit also remained a limiting issue (i.e., CFI = .860; TLI = .845; see Table 1 for a summary).

Further limitations within the available MAIA literature include elements of measurement bias, such as the use of CFA without first conducting EFA [13, 15]; analysing the dimensionality of selected subscales, rather than the full measure [15]; and possible factor over-retention in EFA [10, 12]. Indeed, the criteria for factor retention have not been reported in many cases and others have relied exclusively upon eigenvalues (see Table 1), which has been shown to result in over-retention [27]. Several studies have also compared groups with differing levels of experience with mindfulness and body awareness practices, without first establishing measurement invariance across these levels [6, 13, 26]. This is problematic because individuals with lower levels of experience might encounter greater difficulties in differentiating the different aspects of IA and are likely to be less familiar with some of the terminology, both of which could lead to artefactual results. Relatedly, mean differences in MAIA subscale scores have been examined across gender identity without prior assessment of invariance [28]. Finally, in many cases, translations of the MAIA appear to have been used without any prior examination of factorial validity (e.g. Dutch: [29]; French: [30]; Greek: [31]; Polish: [32]), which is also likely to have resulted in artefactual results [33].

### The present study

The primary aim of the present study was to examine the psychometric properties of a Bahasa Malaysia (Malay) translation of the MAIA in a sample of Malay-speaking adults. We also specified series of smaller procedural aims and associated hypotheses. Firstly, in accordance with best practise guidelines [33, 34], we planned to investigate the Malaysian MAIA factor structure using EFA. EFA facilitates exploration of the underlying factor structure of the data without any *a priori* modelling limitations. This was an important and necessary procedural step because it is possible that Malaysian cultural identities may have an impact upon the construct of IA [35] and the dimensionality of MAIA scores. For example, emerging evidence in the Malaysian context suggests that interoception-related constructs, such as one’s hunger and satiety cues, may be conceptually complex compared to many Western populations [36].

Given the numerous translational MAIA studies that have proposed models with a reduced number of items or factors (see Table 1), we hypothesised that our EFA would indicate a reduced model. Next, we assessed the fit of the EFA-derived model using CFA and compared it with Mehling and colleagues’ 8-factor model, using a separate sample. Given the nature of EFA modelling, we expected that the EFA-derived model would evidence a superior fit to the Malaysian data than the 8-factor parent model. Following this, we estimated internal consistency coefficients for both models. We hypothesised that scores would be internally reliable, with the exception of the Not-Distracting and Not-Worrying subscales from Mehling and colleagues’ 8-factor model [6] (see Table 1). We also planned to examine the invariance of MAIA scores across sex, which would facilitate future comparisons of mean differences in latent variable scores in future studies. We expected that we would be able to demonstrate invariance across configural, metric, and scalar levels. Given that strict invariance is rarely met [37] and acknowledged to be overly restrictive [38], we did not expect to be able to demonstrate invariance at this level.

Finally, we aimed to conduct a preliminary investigation of the convergent validity of MAIA scores in our sample, using existing measures of trait mindfulness and self-esteem. These constructs were selected because they have been previously validated for use with Malay-speaking adults and because they have used to demonstrate convergent validity previously. We expected to find that MAIA scores would be positively associated with trait mindfulness and self-esteem [4, 6, 39, 40]. Taken together, these steps provide a robust examination of the psychometric properties of the MAIA in our sample and allowed us to avoid many of the limiting issues that affect this area of research, as described above.

## Method

### Participants

The sample (*N* = 815) consisted of 403 women and 412 men. All participants were of Malay ancestry, which is the majority ethnic group in Malaysia at present. All Malays are considered Muslim as required by Malaysian constitutional law. Participants were aged between 18 to 69 years (*M* = 33.89, *SD* = 8.80) and in self-reported body mass index (BMI) from 13.26 to 49.86 kg/m^2^ (*M* = 24.82, *SD* = 5.48). In terms of educational qualifications, 32.1% had completed secondary schooling, 39.4% had an undergraduate degree, 18.7% had a postgraduate degree, and the remainder had some other qualification. Of the total sample, 34.6% were single, 62.7% were married, 2.3% were divorced, and 0.4% had some other marital status.

### Questionnaire translation

The MAIA [6] was translated into Bahasa Malaysia, the Malay lect used in Malaysia, following best-practice guidelines for test adaptation [33]. Specifically, we used the 5-stage procedure recommended by Beaton and colleagues [41]. In the first stage, the items, instructions, and response anchors of the MAIA were forward-translated from English to Malay by an informed and an uninformed translator. In a second stage, the two translations were examined by a third independent and blind translator, who resolved discrepancies between the translations and produced a synthesised forward-translation. In a third stage, two new independent and blind translators back-translated the synthesised translation into English [42]. In a fourth stage, the forward- and back-translations were examined by a bilingual committee comprising all aforementioned translators, a methodologist, and the final four authors of the present study (all of whom are bilingual Malaysians). The committee discussed the translations and settled minor word-choice and grammatical issues using a consensual approach, which resulted in a pre-final version of the Malay MAIA. In a final stage, the pre-final version of the MAIA was pre-tested in a sample of 42 Malaysian Malays (women = 54.8%) who approximated the target population. These participants were asked to rate each item for understanding on a 5-point scale (1 = *do not understand at all*, 5 = *understand completely)*. The mean responses per item were then assessed by the committee and were suggestive of overall good understanding (M = 4.24, SD = 0.44). Thus, no further revisions were made to the Malay MAIA items, which we considered to be effectively translated in terms of semantic and item equivalence. Items of the Malay MAIA alongside the English originals are reported in Table 3.

### Additional measures

#### Self-esteem

Self-esteem was assessed using the Rosenberg Self-Esteem Scale (RSES [43]; Malay translation: [44]). The RSES is a 10-item measure which assesses one’s overall sense of self-worth (sample item: “I feel that I have a number of good qualities”). Items were rated on a 4-point scale ranging from 1 (*strongly disagree*) to 4 (*strongly agree*). In its original form, 5 items are reverse-coded, but in the Malay translation one of these reverse-coded items (Item 8) loads negatively. Therefore, following Swami’s [44] recommendation, Item 8 was not reverse-coded in the present study. Higher total RSES scores indicate higher self-esteem. Malay RSES scores have been found to produce a 1-dimensional factor structure, good test-retest reliability (across a 5-week interval), and patterns of convergent and discriminant validity, as well as adequate internal consistency [44]. In the present study, ω was .74 (95% CI = .72, .76).

#### Mindfulness

We assessed trait mindfulness using the 15-item Mindful Attention Awareness Scale (MAAS [45]; Malay translation: [46]). The MAAS assesses the degree to which one is attentive to, and aware of, present moment experiences in everyday activities (sample item: “I find it difficult to stay focused on what’s happening in the present”). MAAS items are rated on a 6-point scale (0 = *almost always*, 5 = *almost never*), and scores were reverse-coded so that higher-scores reflect greater trait mindfulness. Scores on the Malay MAAS produces a 1-dimensional factor structure, with adequate internal consistency and good patterns of convergent validity [46]. In the present study, ω for MAAS scores was .93 (95% CI = .92, .94).

#### Demographics

Participants were also asked to provide demographic details consisting of sex, age, highest educational attainment, marital status, height, and weight. We used the final two items to compute self-reported BMI as kg/m^2^, used here for sample descriptive purposes.

### Procedure

Ethical approval was granted by the Anglia Ruskin departmental ethics committee prior to data collection (approval code: EHPGR-13). Data were collected in March-April 2019 via a Qualtrics^TM^ (www.qualtrics.com) research panel. Study eligibility was limited to citizens of Malaysia, who were of adult age (≥ 18 years), of Malay ancestry, and fluent in Malay. Participants were first required to provide digital informed consent. Following this, the measures described above were completed in a counterbalanced order. During survey completion participants were prompted to answer omitted questions but were still free to leave these blank if they chose to do so. Written debriefing information was provided at the end of the survey and participants were paid AUD 2.00 as remuneration for their time.

### Analytic strategy

We examined IP addresses to ensure that participants did not complete the survey more than once. Improbable BMI values (< 12 or > 50 kg/m^2^) were removed and treated as missing data. After this, inspection of the main data set for missing values revealed a minimal amount of missing data (0.3%) and it was ascertained that all 32 MAIA items had been completed by all participants. The data that were missing were not missing completely at random (MCAR), as determined by Little’s MCAR analyses, χ^2^(5159) = 7888.36, *p* < .001. Therefore, missing values were imputed using the multiple imputation technique using the MICE package [47] in *R* [48].

In accordance with best-practise guidelines [33], we employed a two-step process to examine the factor structure of the Malay MAIA, which involved exploratory factor analysis (EFA) in the first step and confirmatory factor analysis (CFA) in the second [33, 34]. We divided the total sample using a computer-generated random seed to ensure that adequate sample sizes were available for both steps. This process resulted in one split-half sample for EFA (women *n* = 187, men *n* = 190), and a second split-half sample for CFA (women *n* = 216, men *n* = 222). With data from the first split-half, we conducted a principal-axis EFA using the Psych package [49] in *R* [48]. Both sample sizes met Worthington and Whittaker’s item-communality requirements [34], as well as additional assumptions for EFA regarding item distributions, average item correlations, and item-total correlations [50].

We computed the Kaiser-Meyer-Olkin (KMO) measure of sampling adequacy and Bartlett’s test of sphericity to assess whether our data were factorable. Ideally, the KMO should be ≥ .80 [51], and Bartlett’s test of sphericity should be significant. We used a Varimax rotation for the EFA (because of the expectation of multiple, inter-correlated factors) and the number of factors to be extracted was based upon a comparison between eigenvalues in the current dataset and parallel analysis, with only values that are > 1.0 and greater than those from the parallel analysis being retained [27]. Only items with loadings ≥ .33 were retained, in accordance with Comrey and Lee’s recommendation [52].

With data from the second split-half, we conducted CFA using the lavaan [53], semTools [54], and MVN packages [55] in *R* [48]. Proactive Monte Carlo simulations [56] using the SIMsem package [57] in *R* [48] suggested that our sample (*n* = 438) surpassed the minimum requirement 312 for this analysis. Our aim was to test the 8-factor model proposed by Mehling and colleagues [6] and, if discrepant, the model suggested by our EFA results. The data did not meet normal distribution thresholds at either the univariate (Sharipo-Wilks *p* < .001) or multivariate level (Mardia’s skewness = 19007.04, *p* < .001, Mardia’s kurtosis = 113.29, *p* < .001). Therefore, we obtained parameter estimates using the robust maximum likelihood method with the Satorra-Bentler correction [58]. Goodness-of-fit was examined using the following indices: the normed model chi-square (χ^2^/df = χ^2^_normed_), with values < 3.0 indicating good fit [24]; the Steiger-Lind root mean square error of approximation (RMSEA), with values close to .06 indicating good fit, and values up to .08 evidencing adequate fit [59]; the standardised root mean square residual (SRMR), with values < .09 indicating good fit [24]; the comparative fit index (CFI), with values close to or > .95 indicating good fit [24]; the Tucker-Lewis index (TLI), with values close to or > .95 indicating good fit [24]; and Bollen’s Incremental Fit Index (BL89), with values close to or > .95 indicating good fit [24]. To assist in the assessment of model parsimony, we report the Parsimony Goodness-of-Fit Index (PGFI). PGFI is an adjustment to goodness-of-fit that penalises models that are less parsimonious, while not penalising for having more parameters [60]. There are currently no agreed thresholds for PGFI, although Mulaik and colleagues [60] suggest that values should be within .50-.90, and higher values are considered to be indicative of a more parsimonious model [61]. Finally, to compare relative fit across models, the Akaike information criteria (AIC) was computed, with the smallest values indicative of preferable fit [62].

In the second split-half, we also used multi-group CFA [63] to assess measurement invariance at the configural, metric, scalar, and strict levels between women and men. Scholars have argued that the Δχ^2^ statistic is an excessively conservative standard for invariance [64]. Therefore, we also used ΔCFI < .01 as an indicator of metric invariance [65]. We used ΔCFI < .01 and ΔRMSEA < .015 or ΔSRMR < .030 as criteria for scalar invariance [63], although it has also been suggested that ΔCFI < .01 may be satisfactory [65].

We estimated internal consistency using ω [22], which–as previously outlined–is likely to provide a more reliable estimate of internal consistency than Cronbach’s α in the case of the MAIA [21]. Values greater than .70 reflect adequate internal reliability [21]. Convergent validity was examined using the Fornell-Larcker criterion [66], with average variance extracted (AVE) values of ≥ .50 considered adequate [67]. Sex differences in MAIA scores would only be investigated using an independent-samples *t*-test should scalar or partial scalar invariance be established. To assess convergent validity, we estimated the correlations between MAIA subscale scores and scores on the measures of self-esteem and mindfulness.

## Results

### Exploratory factor analysis

We conducted a principal-axis EFA with the data from the first split-half sample (*n* = 377). Bartlett’s test of sphericity was significant, χ^2^(496) = 6796.90, *p* < .001, and KMO = .94, which together indicate that the MAIA items had sufficient common variance for factor analysis. Results from the EFA indicated that there were six factors with λ > 1.0, and inspection of the Scree plot indicated that there was one primary factor, with a steep cut-off to the remaining factors. The results of parallel analysis suggested that three factors from the actual data had λ greater than the criterion λ generated from the random data (*i*.*e*., λ_1_ 12.77 > 1.59, λ_2_ 2.24 > 1.51, λ_3_ 1.84 > 1.44). The remaining three factors had an λ that was lower than the corresponding criterion λ generated from the random data (i.e., λ_4_ 1.25 < 1.40, λ_5_ 1.02 < 1.35, λ_6_ 1.01 < 1.32). Based upon the results of the parallel analysis, we retained three factors in this subsample, which explained 53.0% of the common variance. Factor loadings are reported in Table 2.

**Table 2:** Multidimensional Assessment Of Interoceptive Awareness (MAIA) items in english and (in Italics) in Bahasa Malaysia (Malay) and associated item-factor loadings from the first split-half subsample.

Item	Dimension in Mehling et al. (2012)	F1	F2	F3
1. When I am tense, I notice where the tension is located in my body. / *Apabila saya rasa ketegangan*, *saya dapat rasa di mana ketegangan tersebut berada dalam badan saya*.	NT	.24	.19	.08
2. I notice when I am uncomfortable in my body. / *Saya perasan apabila saya rasa tidak selesa dengan badan saya*.	NT	.16	.22	.10
3. I notice where in my body I am comfortable. / *Saya perasan bahagian mana pada badan saya yang saya rasa selesa*.	NT	.25	**.40**	.19
4. I notice changes in my breathing, such as whether it slows down or speeds up. / *Saya perasan perubahan pada pernafasan saya*, *seperti semakin perlahan atau semakin cepat*.	NT	.16	.**41**	14
5. I do not notice (I ignore) physical tension or discomfort until they become more severe. / *Saya tidak perasan (saya abaikan) ketegangan fizikal atau ketidakselesaan sehingga ia menjadi lebih parah*.	ND	.06	.09	-.20
6. I distract myself from sensations of discomfort. / *Saya boleh alihkan perhatian saya daripada sensasi ketidakselesaan*.	ND	**.39**	.31	.04
7. When I feel pain or discomfort, I try to power through it. / *Apabila saya rasa sakit atau tidak selesa*, *saya cuba mengharunginya*.	ND	.25	**.41**	.12
8. When I feel physical pain, I become upset. / *Apabila saya rasa sakit secara fizikal*, *saya jadi kecewa*.	NW	.07	.07	.05
9. I start to worry that something is wrong if I feel any discomfort. / *Saya mula risau bahawa ada sesuatu yang tidak kena jika saya rasa tidak selesa*.	NW	.19	.23	.18
10. I can notice an unpleasant body sensation without worrying about it. / *Saya dapat perasan sensasi badan yang tidak menyenangkan tanpa merisaukannya*.	NW	**.26**	.10	-.01
11. I can pay attention to my breath without being distracted by things happening around me. / *Saya boleh memberi perhatian kepada pernafasan saya tanpa terganggu oleh perkara-perkara yang berlaku di sekeliling saya*.	AR	**.60**	.18	.24
12. I can maintain awareness of my inner bodily sensations even when there is a lot going on around me. / *Saya dapat kekalkan kesedaran pada sensasi badan dalaman saya walaupun banyak perkara sedang berlaku di sekeliling*.	AR	**.63**	.17	.29
13. When I am in conversation with someone, I can pay attention to my posture. / *Apabila saya berbual dengan seseorang*, *saya boleh beri perhatian kepada postur saya*.	AR	**.59**	.17	.28
14. I can return awareness to my body if I am distracted. / *Saya boleh kembalikan kesedaran kepada badan saya jika saya terganggu*.	AR	**.67**	.27	.17
15. I can refocus my attention from thinking to sensing my body. / *Saya boleh memfokuskan perhatian saya daripada berfikir kepada sensasi badan*.	AR	**.73**	.25	.18
16. I can maintain awareness of my whole body even when a part of me is in pain or discomfort. / *Saya dapat kekalkan kesedaran pada seluruh badan saya walaupun sebahagiannya sakit atau tidak selesa*.	AR	**.65**	.18	.15
17. I am able to consciously focus on my body as a whole. / *Saya dapat fokus secara sedar kepada keseluruhan tubuh saya*.	AR	**.53**	.30	**.41**
18. I notice how my body changes when I am angry. / *Saya perasan bagaimana tubuh saya berubah apabila saya marah*.	EA	.25	**.53**	.19
19. When something is wrong in my life I can feel it in my body. / *Apabila ada sesuatu yang tidak kena dalam hidup saya*, *saya dapat merasakannya dalam badan saya*.	EA	.29	**.48**	.12
20. I notice that my body feels different after a peaceful experience. / *Saya perasan bahawa badan saya rasa berbeza selepas pengalaman yang menenangkan*.	EA	.24	**.64**	.22
21. I notice that my breathing becomes free and easy when I feel comfortable. / *Saya perasan pernafasan saya menjadi tenang dan mudah apabila saya rasa selesa*.	EA	.18	**.72**	.22
22. I notice how my body changes when I feel happy or joyful. / *Saya perasan bagaimana tubuh saya berubah apabila saya rasa gembira*.	EA	.19	**.66**	.31
23. When I feel overwhelmed I can find a calm place inside. / *Apabila saya rasa tertekan*, *saya dapat mencari ketenangan dalaman*.	SR	**.37**	.18	.30
24. When I bring awareness to my body I feel a sense of calm. / *Apabila saya membawa kesedaran kepada badan saya*, *saya rasa tenang*.	SR	**.41**	**.47**	**.44**
25. I can use my breath to reduce tension. / *Saya boleh gunakan pernafasan saya untuk mengurangkan ketegangan*.	SR	**.44**	.22	**.47**
26. When I am caught up in thoughts, I can calm my mind by focusing on my body/breathing. / *Apabila saya terlalu berfikir*, *saya dapat tenangkan minda dengan memberi fokus kepada badan/pernafasan saya*.	SR	.**47**	.05	**.41**
27. I listen for information from my body about my emotional state. / *Saya mencari maklumat daripada badan saya mengenai keadaan emosi saya*.	BL	.31	.18	.25
28. When I am upset, I take time to explore how my body feels. / *Apabila saya kecewa*, *saya mengambil masa untuk meneroka bagaimana badan saya merasainya*.	BL	.24	.17	.20
29. I listen to my body to inform me about what to do. / *Saya mendengar isyarat badan saya untuk memberitahu saya apa yang perlu dilakukan*.	BL	.29	.17	.23
30. I am at home in my body. / *Saya rasa selesa dengan badan saya*	TR	.22	.26	**.63**
31. I feel my body is a safe place. / *Saya rasa badan saya adalah tempat yang selamat*.	TR	.28	.20	**.69**
32. I trust my body sensations. / *Saya mempercayai sensasi badan saya*	TR	.23	.27	**.70**

Items in bold indicate items associated with each factor. NT = Noticing, ND = Not-Distracting, NW = Not-Worrying, AR = Attention Regulation, EA = Emotional Awareness, SR = Self-Regulation, BL = Body Listening, TR = Trusting, F = Factor.

### Factor interpretation and further analyses

Nine items did not load onto any of the three factors (Items 1, 2, 5, 8, 9, 10, 27, 28, and 29) and four items showed cross-loadings > .33 (Items 17, 24, 25, and 26). Therefore, these items were discarded from analyses. Items that loaded on the first factor included six Attention Regulation items (Items 11, 12, 13, 14, 15, and 16) and two additional items that were consistent with the ability to sustain and control attention to bodily sensations (Items 6 and 23). Accordingly, we continued to refer to this factor as Attention Regulation, and ω for scores on this factor was .88 (95% CI = .86, .91). Items that loaded onto the second factor included all of the Emotional Awareness items (Items 18, 19, 20, 21, and 22), two of the Noticing items (Items 3 and 4), and one of the Not-Distracting items (Item 7). Overall, the items on this factor primarily reflect to the tendency to notice bodily sensations, and, secondly, the awareness of how the body changes with emotional states. We, therefore, referred to this factor as Bodily and Emotional Awareness, and ω for scores on this factor was .88 (95% CI = .85, .91). Finally, the third factor included all three Trusting items (Items 30–32), and ω for scores on this factor was .85 (95% CI = .80, .88).

### Confirmatory factor analyses and measurement invariance

In the second split-half sample (*n* = 438), we first examined the fit of Mehling and colleagues’ 32-item, 8-factor model [6]. While some indices were adequate, others were below acceptable levels: SBχ^2^(436) = 799.75, SBχ^2^_normed_ = 1.83, robust RMSEA = .055 (90% CI = .049-.061), SRMR = .054, robust CFI = .927, robust TLI = .917, BL89 = .900, AIC = 40274.51, PGFI = .695. Modification indices were, therefore, consulted to improve model fit, with modifications being based on correlations among similar items from the same factor, and in accordance with results from likelihood ratio tests. Specifically, error covariances were successively freed between Items 18 and 19 [MI = 51.1; χ^2^(1) = 51.11, *p* < .001], 21 and 22 [MI = 22.8; χ^2^(1) = 22.48, *p* < .001], and 9 and 10 [MI = 17.8; χ^2^(1) = 20.33, *p* < .001]. These modifications resulted in an improved model fit, although values for CFI, TLI, and BL89 were still less than ideal: SBχ^2^(433) = 748.15, SBχ^2^_normed_ = 1.73, robust RMSEA = .051 (90% CI = .045-.057), SRMR = .052, robust CFI = .937, robust TLI = .928, BL89 = .911, AIC = 40186.59, PGFI = .700. The standardised estimates of factor loadings ranged from .48 to .89. Internal consistency coefficients were greater than .82 for all subscale scores except Not-Distracting (ω = .62, 95% CI = .54, .68) and Not-Worrying (ω = .68, 95% CI = .62, .73). Convergent validity for this model was less-than-adequate, because while AVE was greater than .50 for six subscales, values for the Not-Distracting and Not-Worrying subscales were low (.34 and .44, respectively).

**Fig 1:**
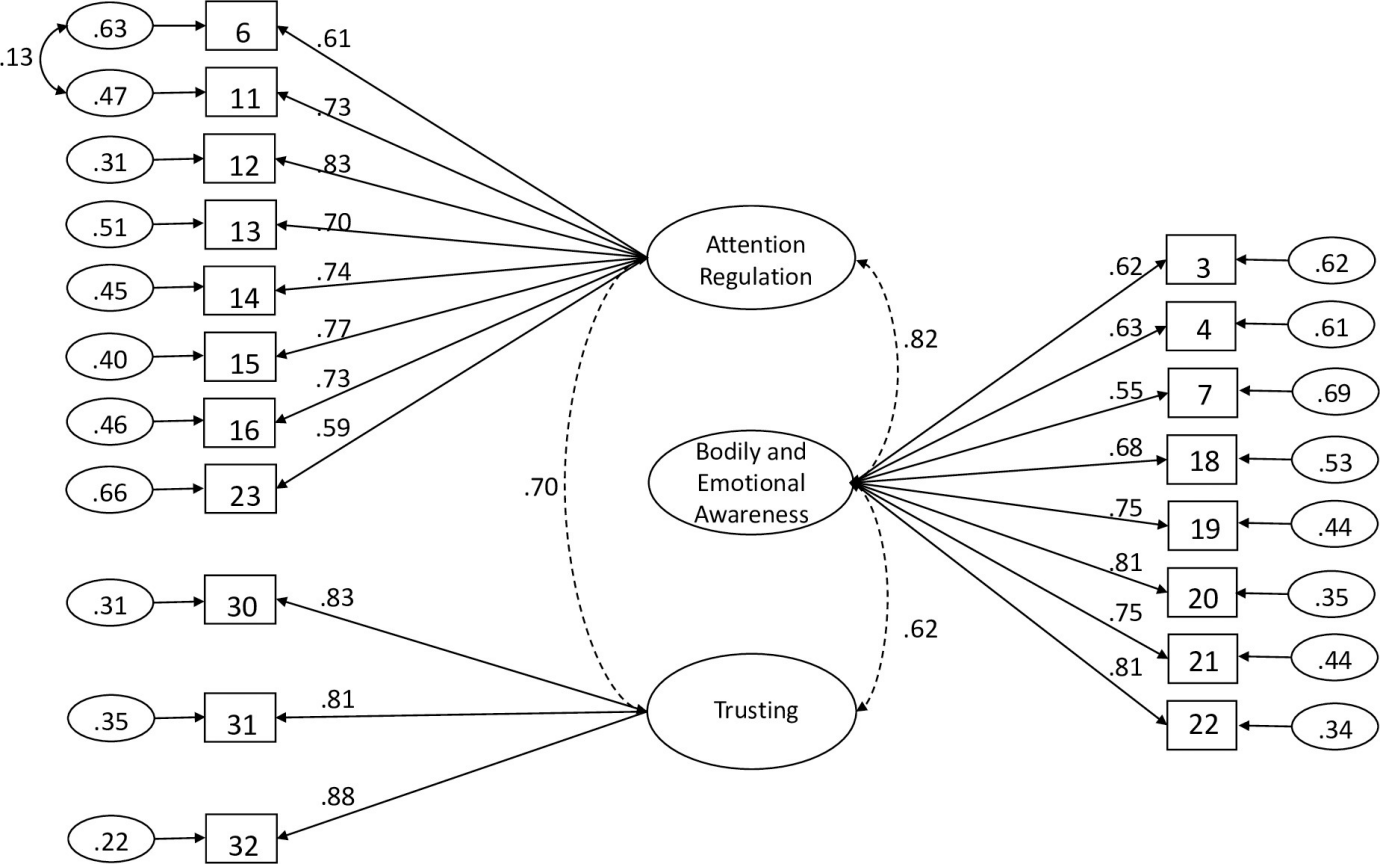
Path diagram and estimates for the 3-dimensional model of Multidimensional Assessment of Interoceptive Awareness scores. The large ovals represent the latent constructs, with the rectangles representing measured variables, and the small circles with numbers representing the residual variables (variances). The path factor loadings are standardised with significance levels were determined by critical ratios (all p < .001).

Next, we examined the fit of the EFA-derived, 3-factor model. Again, some indices were adequate, but others were below ideal levels: SBχ^2^(149) = 283.33, SBχ^2^_normed_ = 1.90, robust RMSEA = .060 (90% CI = .049-.071), SRMR = .045, robust CFI = .948, robust TLI = .940, BL89 = .943, AIC = 23535.79, PGFI = .698. Therefore, modification indices were consulted to improve model fit. Error covariances were freed between Items 6 and 11, which both refer to the maintenance of attention to bodily sensations [MI = 6.1; χ^2^(1) = 6.05, *p* = .014]. This resulted in an improved model fit: SBχ^2^(148) = 280.86, SBχ^2^_normed_ = 1.90, robust RMSEA = .060 (90% CI = .049-.071), SRMR = .045, robust CFI = .949, robust TLI = .941, BL89 = .944, AIC = 23531.75, PGFI = .695. The standardised estimates of factor loadings ranged from .55 to .88 (see Fig 1). In this sample, ω for scores on each factor were as follows: Attention Regulation = .89 (95% CI = .87, .91), Bodily and Emotional Awareness = .88 (95% CI = .85, .91), Trusting = .88 (95% CI = .84 .91). Convergent validity for this model was less-than-adequate, because, while AVE was .70 for Trusting, values for Attention Regulation and Bodily and Emotional Awareness were below .50 (.47 and .49, respectively).

While both models had an acceptable fit across some indices, both were less than adequate across CFI, TLI, and BL89. Comparison of AIC values indicated that the 3-factor model had substantially better fit, and comparison of PGFI values indicated that the 3-factor model was also more parsimonious. Next, we assessed the 3-factor and 8-factor models for measurement invariance across sex in the second-split half subsample. As can be seen from Table 3, full strict invariance was supported across all relevant indices for both models. Examination of between-group differences across sex (Table 4) revealed small (*d ≤* .18) differences for all variables, however, none were statistically significant after applying the Bonferroni correction (.05/5 = .01).

**Table 3:** Measurement invariance across sex in the second split-half subsample for the 3-factor and 8-factor models.

	Model	SBχ^2^	*df*	Robust CFI	Robust RMSEA	SRMR	Model Comparison	ΔSB*χ*^2^	ΔRobust CFI	ΔRobust RMSEA	ΔSRMR	Δ*df*	*p*
3-factor model	Baseline–women	221.25	149	932	.058	.056							
	Baseline–men	233.93	149	.938	.071	.055							
	Configural	456.94	298	.939	.065	.053							
	Metric	471.19	314	.936	.062	.056	Configural vs metric	14.25	.003	.003	.003	16	.927
	Scalar	491.43	330	.934	.061	.057	Metric vs scalar	20.24	.001	.001	.001	16	.399
	Strict	496.15	349	.940	.057	.057	Scalar vs strict	4.72	.006	.004	< .001	19	.913
8-factor model	Baseline–women	653.69	436	.908	.057	.067							
	Baseline–men	666.79	436	.913	.064	.060							
	Configural	1321.89	872	.911	.060	.061							
	Metric	1342.44	896	.912	.059	.064	Configural vs metric	20.55	.001	.001	.003	24	.941
	Scalar	1376.33	920	.911	.059	.064	Metric vs scalar	33.89	.001	< .001	< .001	24	.130
	Strict	1387.01	952	.914	.057	.064	Scalar vs strict	10.68	.003	.002	< .001	32	.856

SB = Satorra-Bentler; CFI = Comparative fit index; RMSEA = Steiger-Lind root mean square error of approximation; SRMR = standardised root mean square residual.

**Table 4:** Bivariate correlations between attention regulation, bodily and emotional awareness, and trusting for women (Top Diagonal) and men (Bottom Diagonal).

		(1)	(2)	(3)	(4)	(5)
(1) Attention Regulation			.58**	.50**	.06	.17**
(2) Bodily and Emotional Awareness		.72**		.56**	-.01	.23**
(3) Trusting		.60**	.53**		.15**	.40**
(4) Trait Mindfulness		.21**	.07	.30**		.45**
(5) Self-Esteem		.07	.12*	.34**	.08	
Women	*M*	3.25	3.80	3.85	3.85	30.75
	*SD*	1.03	0.83	1.10	0.97	4.96
Men	*M*	3.24	3.63	3.73	3.99	30.08
	*SD*	0.84	1.04	1.00	1.04	4.79
	*T*	-0.10	-2.45	-1.72	2.08	-1.96
	*P*	.922	.015	.085	.038	.050
	*D*	0.01	0.18	0.11	0.14	0.14

*p < .05

**p < .001.

### Convergent validity

An examination of the convergent validity of the 3-dimensional MAIA scores was conducted by computing bivariate correlations with scores on all additional measures included in the present study. These analyses were conducted separately for men and women, using the total sample (see Table 4). For both women and men, there were moderate-to-strong inter-correlations for scores on the three MAIA subscales. In men, there were weak, positive associations between scores on the MAIA subscales and scores for trait mindfulness and self-esteem. However, associations between Attention Regulation and self-esteem, and Bodily and Emotional Awareness and trait mindfulness did not reach statistical significance. In women, all scores for all three MAIA subscales had significant, positive associations with self-esteem, but only scores for Trusting were significantly associated with trait mindfulness.

## Discussion

The aim of the present study was to assess the psychometric properties of MAIA scores in a sample of Malaysian Malay adults. The results from our EFA indicated that MAIA scores reduced to three factors, with a total of 19 items. The EFA-derived model was compared with the 8-factor parent model [6] using CFA. We found that both models had a good fit on some indices, but a less-than-ideal fit on others. After successively freeing error covariances for theoretically similar items in both models, the 3-factor model demonstrated better fit comparatively. Further analyses revealed that all factors from our EFA-derived model had satisfactory levels of internal consistency. Finally, both models were demonstrated to be fully invariant across sex.

There was a limited degree of similarity between the parent model and our EFA-derived model. The primary factor from our final model comprised 6 of the 7 items from the original Attention Regulation subscale, in addition to Item 6 from the parent Not-Distracting subscale and Item 23 from the parent Self-Regulation subscale. Although this is a unique grouping when considered against the available literature, both additional items appear to fit well theoretically (i.e., both refer to the regulation of attention toward interoceptive stimuli). It is somewhat surprising that Item 6 was found to load positively on this factor, given that it is reverse scored within the parent model. It is possible that this difference reflects cultural values that are specific to Malaysian Malays. For example, the social ideal of *senang hati*, which refers to an untroubled, relaxed state of mind (for an overview see [68]) may explain why the tendency to distract attention from sensations of discomfort appears to be regarded as a positive attribute in the present sample.

The second factor from our EFA-derived model was a combination of all the Emotional Awareness items from the parent model, two of the Noticing items (Items 3 and 4), and one of the Not-Distracting items (Item 7). All items refer primarily to the tendency to notice bodily sensations and secondly to the awareness of associations between bodily sensations and emotional states. There is some precedent for the combination of the two facets of IA in two studies that sought to reduce the number of MAIA subscales [19, 20], and Item 4 has been previously associated with Emotional Awareness in two translational studies [10, 12]. Furthermore, the Emotional Awareness and Noticing subscales were also combined in the Japanese translation of the MAIA [16, 18]. There are a number of explanations for these cross-study findings. One possibility is that the awareness of changes in breathing could be tapping an awareness of anxiety-related states. It could also be the case that the findings from the present study (and the work of Fujino [16], and Shoji and colleagues [18]), reflect the lesser distinction between bodily sensations and emotional processes in Asian samples relative to Western samples [35]. For example, research suggests that Asian participants tend to demonstrate a greater emphasis on bodily states when describing an emotional experience and tend to perceive bodily and psychological states as interconnected [35].

The third factor from our EFA-derived model comprised all three items from the original Trusting subscale, which is consistent with the available literature. Indeed, as can been seen from Table 1, the Trusting subscale has been included in all of the available MAIA models and levels of internal consistency have been consistently satisfactory, despite the small number of items.

Therefore, while IA facets of Trusting, Attention Regulation, Emotional Awareness and Noticing from Mehling and colleagues’ multidimensional conceptual model [6] are all represented to some extent within our EFA-derived model, the facets of Not-Distracting, Not-Worrying, Body Listening, and Self-Regulation are not. It is notable that some loss of facet and item coverage is common in test adaptation studies with Malay participants (e.g., [36, 69]. It is possible that the results of the present study reflect the fact that the MAIA is based on a Western model of interoception that may not be fully applicable to non-Western samples. For example, as previously discussed, it is possible that the distinction between bodily sensations and emotional processes is lesser for Eastern samples relative to western samples [35]. Similarly, there are cultural values specific to Malaysian Malays such as *maruah* (which refers to a sense of dignity or pride regarding both self-perceptions, and what others think about the individual [68]), and the aforementioned concept of *senang hati*, which could both impact the conceptualisation of IA in Malaysia. To address this issue, future researchers could adopt an emic approach in order to better understand the dimensionality of interoception in Malaysian adults [70]. Such research could be initiated with the use of qualitative techniques.

Nevertheless, while our EFA-derived model is currently unique in terms of the relatively high number of items and factors that have been excluded, it is worth noting that several other authors have also failed to replicate the parent structure [14, 15, 17, 18, 71]. Furthermore, it is arguable that many of the extant 8-factor models have retained factors or items erroneously. For example, several studies have retained subscales with fewer than three items [10–12] and retained items with large cross-loadings [12, 26]. It is also worth noting that the factors that were excluded from our EFA-derived model have been commonly problematic within the available literature. For example, as previously highlighted, the Not-Distracting and Not-Worrying subscales have either been altered or excluded from the majority of the available validation studies [10–18, 26, 71]. Similarly, the Body Listening and Self-Regulation subscales have been either discarded or altered in many cases [7, 10, 13, 14, 17, 18].

While our model demonstrated satisfactory levels of internal consistency and full invariance across sex, we encountered difficulties when assessing convergent validity. In particular, we were constrained by the paucity of measures that have been validated for use with Malaysian adults. The preliminary evidence that we present here is indicative of adequate convergent validity for the Trusting subscale. We were also able to provide adequate evidence of convergent validity for the Trusting subscale, as assessed using the Fornell-Larker criterion [66]. However, for the two remaining subscales our results do not support convergent validity. We were also surprised by the lack of significant associations between the Bodily and Emotional Awareness subscale and trait mindfulness (for both sexes), and between the Attention Regulation subscale and trait mindfulness for women. Given that the MAIA was developed with the intention to assess mindful body awareness [4, 6, 26], the subscales should theoretically be associated with trait mindfulness, as demonstrated with previous versions of the measure (for an overview, see [4]). We, therefore, advise future researchers to use the Attention Regulation and Bodily and Emotional Awareness subscales with caution, and, ideally, to conduct further assessments of construct and convergent validity with a wider range of measures once they have been validated for use in Malay-speaking populations. Additional issues that also warrant greater attention in future studies include examination of test-retest reliability; examination of the Malay MAIA across other Malaysian ethnic groups, and; recruitment of a larger sample, which would facilitate the replication of the work with greater certainty in the stability of the correlational results and our EFA results. Finally, future researchers should also seek to translate and examine the MAIA-2 [26], which contains five additional items.

Despite these limitations, the present work provides important evidence regarding the dimensionality of MAIA scores. Given the paucity of measures that have been validated for use in Malay-speaking populations, the Malay MAIA will be a useful contribution to knowledge, particularly for researchers seeking to examine the construct of interoception in Malaysia, which has received little attention to date. We recommend that researchers should include all 32 translated items and examine (and report) the properties of both the 8 and 3-factor structures. Regarding the MAIA more generally, the challenges encountered in the present work mirror the accounts from previous psychometric assessments of the MAIA, and we therefore further encourage researchers to reassess the dimensionality of MAIA scores any time the measure is used.
